# Effect of MALAT1 Polymorphisms on Papillary Thyroid Cancer in a Chinese Population

**DOI:** 10.7150/jca.28887

**Published:** 2019-09-19

**Authors:** Jing Wen, Liang Chen, Hua Tian, Ji Li, Miao Zhang, Qing Cao, Wei Zhang, Shi Chen, Lixin Shi

**Affiliations:** 1 Department of Ultrasonics, the Affiliated Hospital of Guizhou Medical University, Guiyang 550004, China;; 2 Department of Pathophysiology, the Institute of Basic Medicine, Guizhou Medical University, Guiyang 550004, China;; 3 Department of General Surgery, Wujiang NO.1 People's Hospital, Suzhou 215200, China;; 4 Department of acute infectious disease Prevention, Jiangsu Provincial Center for Disease Control and Prevention, Nanjing 210009, China;; 5 Central Laboratory, the Affiliated Hospital of Guizhou Medical University, Guiyang 550004, China;; 6 Department of Endocrinology, the Hospital Affiliated to Guizhou Medical University, Guiyang 550004, China;; 7 College of Medicine, Henan University of Science and Technology, Luoyang 471023, China; 8 Department of Thyroid Surgery, the Affiliated Hospital of Guizhou Medical University, Guiyang 550004, China;; 9 Department of Public Health Sciences, University of North Carolina Charlotte, Charlotte, NC 28223, USA.

**Keywords:** thyroid cancer, long noncoding RNA, MALAT1, polymorphism, susceptibility.

## Abstract

**Background:** Long noncoding RNA MALAT1 has been previously reported in the carcinogenesis of several tumors, and its potential functional polymorphisms have also been investigated in various diseases. However, the relationship between these polymorphisms and the susceptibility of thyroid cancer has still been largely unknown. In the present study, we aimed to explore the association between MALAT1 polymorphisms and thyroid cancer (TC) susceptibility, as well as potential biological function in TC.

**Methods:** We conducted a case-control study with 1134 papillary thyroid cancer (PTC) patients and 1228 controls to evaluate the potential correlation between MALAT1 genetic variations (single nucleotide polymorphism, SNP) and the risk of PTC. More detailed molecular mechanisms were explored by luciferase assay, cell counting kit-8 (CCK-8), and flow cytometry.

**Results:** MALAT1 SNP rs619586 was identified as a significantly protective factor of PTC susceptibility (*P* = 0.017, OR= 0.76, 95%CI = 0.60-0.95). Further functional experiments of rs619586 indicated that G allele of rs619586 could significantly decrease MALAT1expression, reduce PTC proliferation, and directly increase PTC apoptosis.

**Conclusions:** Our findings suggested that MALAT1 SNP rs619586 could serve as a potential indicator for PTC susceptibility and pathogenesis.

## Introduction

Thyroid cancer (TC) is the most common endocrine malignancy and its incidence has been increasing worldwide over the past decades 1. TC could be classified into papillary thyroid carcinoma (PTC), follicular thyroid carcinoma (FTC), medullary thyroid carcinoma (MTC), and anaplastic thyroid carcinoma (ATC), according to their histological characteristics 2. Over 90% of TC cases are PTC and FTC, together known as the well-differentiated thyroid cancer (WDTC). WDTC patients usually have favorable prognosis, and surgery and radio-iodinated therapy are effective treatment for WDTC. The other cases, however, might eventually dedifferentiate, become aggressive and result in fatality 3.

Both genetic and epigenetic alternations have been explored and identified as the potential triggers or regulators of TC pathogenesis, including single nucleotide polymorphisms (SNPs). SNP is a kind of mispairing or mutation in the genome, comprising at least 1% of the total population 4. Some of SNPs are functional (located in both coding and noncoding regions of the gene) and influence corresponding gene expression via direct or indirect pathways 5-8. In TC, the phosphatidylinositol-3-kinase (PI3K)/Akt and RAS/RAF/MEK/ERK are the crucial signaling pathways, closely associated with the most genetic events during TC occurrence and development. These pathways also play an important role in regulation of cell growth, apoptosis, and survival 9-11. Besides these functional gene-regulatory polymorphisms, other SNPs located in non-coding RNAs (ncRNAs) also draw more attention 12-14.

As an important member of ncRNAs, long non-coding RNAs (lncRNAs) have been identified to involve in most processes of tumorigenesis, including cell growth, survival and metastasis. LncRNAs are usually composed with over 200 nucleotides, and they cannot code or translate to the proteins as the final product 15, 16. Also, lncRNAs generally have relatively lower expression quantity, and often dwell in the nucleus. The metastasis-associated lung adenocarcinoma transcript-1 (MALAT1) is an 8779 bp transcript 17, which has been reported as a crucial factor in several human diseases, including TC. Also, genetic variants of MALAT1, such as SNPs, have been reported to associate with susceptibility to these diseases 18, 19.

In this study, we have investigated whether certain SNPs on MALAT1 could be potential diagnostic biomarkers for PTC. We evaluated the association between MALAT1 tagSNPs and PTC risk, compared MALAT1 expression levels among different genotypes of these SNPs, and analyzed the functional effects of these SNPs on papillary thyroid carcinogenesis.

## Methods and materials

### Ethic statement

This study was approved by the Institutional Ethics Committee of the Hospital Affiliated to Guizhou Medical University (No.201674). The corresponding methods were carried out in accordance with the approved guidelines. All participants in this program received a detailed content of this research and signed a written informed consent form before donating their biological samples.

### Study population and participants

We enrolled 1140 PTC patients and 1230 cancer-free controls from the Hospital affiliated to Guizhou Medical University and the Tumor Hospital of Guizhou Province, China, between April 2012 and March 2016. Detailed participants' demographic and clinical information (including age, gender, topography, lymph node, metastasis, and grade) were provided in table 1 20. Age and gender were well matched and indistinguishable between PTC and control groups (*P* = 0.754 for age and *P* = 0.846 for gender, respectively). Among of 1140 PTC patients, there were 64 participants who donated their tumor and adjacent normal tissue for the further investigation.

### SNP selection

Potential candidate SNPs on MALAT1 were selected according to the following criteria: 1) locating in MALAT1; 2) acting as tagSNPs; 3) minor allele frequency of each SNP exceeding5% in Han Chinese population; 4) A linkage disequilibrium value of *r^2^*< 0.8 for each other. We also added previously reported SNPs on MALAT1 into this study. Four candidate SNPs met these requirements. However, further investigation in Haploview software and 1000 Genomes project demonstrated that the SNPs rs619586 and rs664589 were in complete linkage equilibrium in Chinese. This was confirmed by our genotyping as well. Based on further functional predictions of these two SNPs, we finally includedSNPrs619586 in our study because of its potential regulation in MALAT1 expression.

### DNA extraction and polymorphism genotyping

5 ml peripheral venous blood was collected from each participant. QIAcube HT Plasticware and QIAamp 96 DNA QIAcube HT Kit (Qiagen, Dusseldorf, Germany) were used for DNA extraction, following the manufacturer's protocol. The quality of DNA samples was evaluated based on the corresponding analysis on Nanodrop-2000 spectrophotometer (Thermo, Waltham, MA, USA). Genotyping of lnc MALAT1 polymorphisms was performed in TaqMan SNP Genotyping Assay using ABI Fast 7900HT real-time PCR system (Applied Biosystems, Foster City, CA, USA). The primer were synthesized and applied by Biolight Tec. (Nanjing, Jiangsu, China), and all primer and probe sequences were listed in the Supplementary table s1. SDS 2.4 software (Applied Biosystems) was used for allelic discrimination. Six samples were placed in each plate as the quality control. Additional10% samples were randomly chosen to repeat the genotyping, and the results were 100% consistent.

### RNA isolation and real-time PCR

Total RNA was isolated using TRIzol reagent (Invitrogen, Carlsbad, CA, USA) and then reverse transcribed. The resulting cDNA was used for qPCR in TaqMan Gene Expression Assays (Applied Biosystems) to evaluate the expression of MALAT1 in PTC tissues, and beta-actin was used for normalization as an endogenous control. The results were averaged from three replicates under the same operation and condition.

### Cell culture

Culture condition of TPC-1 was described as our previous study 1. And the BCPAP cell line was purchased from American Type Culture Collection (ATCC, Manassas, VA, USA), and was grown in Dulbecco's Modified Eagle's medium with 10% fetal bovine serum. Both cell lines were maintained at 37°C in a 5% CO_2_-O_2_ mixture.

### Plasmids construction and transfection

The total MALAT1 gene (from NC_000011.10) was synthesized and constructed into pcDNA3.1 vector (Invitrogen) by Biolight Tec. Company (Nanjing, China) as the MALAT1 expression plasmid, and the single site mutation was used to obtain the mutation plasmid with G allele on rs619586 site. For luciferase reporter plasmids, 1000bp fragments of MALAT1 containing A>G rs619586 polymorphism (500bp upstream and 500bp downstream of rs619586) were synthesized and constructed into pGL3-basic vector(Promega, Madison, WI, USA) by Biolight Tec Company (Nanjing, China). All constructs were confirmed by DNA sequencing.

For transfection experiments, 1×10^6^ cells were seeded in each well of a 24-well culture plate. The lipofectamine-2000 transfection reagent (Invitrogen) was used to transfect 0.5μg constructed MALAT1 expression plasmids with different alleles (A and G) on rs619586 site into each well. The eGFP plasmid was co-transfected as the index of transfection efficiency and the internal standard.

### Cell proliferation

Cell proliferation was quantified by cell counting kit-8 (CCK-8). Cells were cultured in a 96-well plate for transfection with MALAT1 wild type and mutation expression plasmids. After transfection and incubation, CCK-8 reagent was added into each well and incubated at 37°C for 2 hours. Absorbance of each well was evaluated at 450nm with a microplate reader. The results were averaged from triplicated readings under the same operation and condition.

### Cell apoptosis assay

Apoptosis was detected by flow cytometry with Annexin V-FITC/propidium iodide (PI) double staining. Because both TPC-1 and BCPAP cell lines did not display the trend of severe apoptosis, we added the cisplatin as the pretreatment to induce the corresponding cellular process. After 24h treatment with MALAT1 expression plasmid and its variants, cells were collected by centrifugation and re-suspended. Annexin V-FITC and PI were then added. Flow cytometer (Becton Dickinson, CA, USA) was used to analyze cell apoptosis and calculate the apoptosis rate. Three replications were performed under the same operations and conditions.

### Statistical analysis

Statistical analyses were performed in SAS software (version 9.13, SAS Institute Inc, Cary, NC, USA). Differences between groups were analyzed using Student's *t*-test. Genotype distribution was analyzed by chi-square test to compare with the Hardy-Weinberg equilibrium. Differences between the distributions of demographic and clinical characteristics in PTC patient and control groups were evaluated by Student's *t*-test or chi-square tests. Logistic regression was performed to analyze the association between MALAT1 polymorphisms and TC risk, adjusted by age, gender, topography, lymph node, metastasis, and tumor grade.

## Results

### Associations between MALAT1 polymorphism and PTC risk

The distributions of demographic and clinical characteristics of the participants were listed in the Table 1. There were no significant differences in gender and age between TC patient and control groups. For TC patients, 78.0%, 12.2%, 8.6% and 1.2% were diagnosed as T1, T2, T3, and T4, respectively. For lymph node metastasis, 61.3% patients were in N0 (with no lymph node metastasis) and the remaining 38.7% were in N1. Furthermore, only 1.8% of TC patients demonstrated distant metastasis in their pathological examinations. In addition, 84.9% patients were diagnosed as early stage (I+II) and 15.1% were in advanced stage (III+IV).

Data from SNP genotyping assays for MALAT1 polymorphisms were shown in table 2. There was statistically significant association between SNP rs619586 and PTC. In the additive model, genotype AG+GG of rs619586showed a protective effect compared with genotype AA (*P* = 0.017, OR = 0.76, 95%CI = 0.60-0.95). Besides, the dominant and recessive model also displayed their significances in PTC patients (*P*= 0.017, OR = 0.76, 95%CI = 0.60-0.95 for dominant and *P*= 0.033, OR = 0.11, 95%CI =0.01-0.84 for recessive model, respectively). There was no significant association between either rs11227209 or rs3200401 and the risk of PTC in the participants.

### Stratified analysis of MALAT1 polymorphism in clinical features in PTC

Stratified analysis results were shown in table 3. SNP rs619586 also displayed its significantly protective role in the group with age ≤ 45(*P* = 0.035, OR = 0.70, 95%CI = 0.50-0.98), male group (P = 0.036, OR = 0.61, 95%CI = 0.39-0.87), and M0 group (patients with no distant metastasis) (P = 0.036, OR = 0.77, 95%CI = 0.60-0.98).

### The genotype-phenotype relationship between MALAT1 expression and its polymorphism

**Figure 1:**
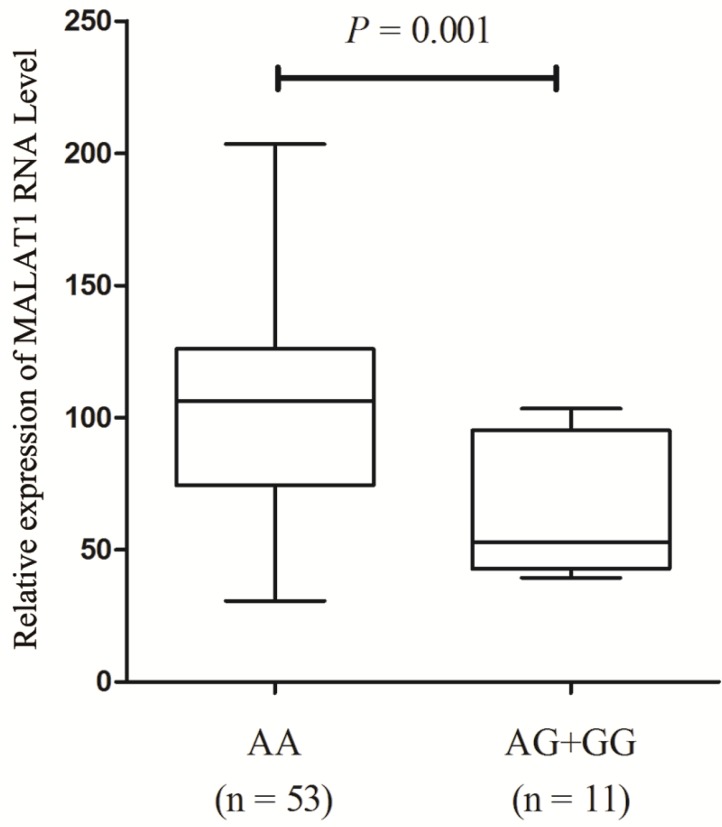
Expression of lnc MALAT1 in PTC tissues with different genotypes of rs619586.

We further quantified MALAT1 expression levels in PTC tissue samples with different genotypes of SNP rs619586 in order to investigate the influence of rs619586 variants on MALAT1 transcription. As displayed in figure 1, after standardization by each corresponding normal tissues, the MALT1 expression in patients' tumor tissues displayed higher expressions (Ranging from 5.87 to 201.6 folds). And MALAT1 expression level in samples with AA genotype was significantly higher than those with AG+GG genotypes (*P* = 0.001, fold change = 1.65).

### The effects of MALAT1 polymorphism on MALAT1 transcription in functional analysis

**Figure 2:**
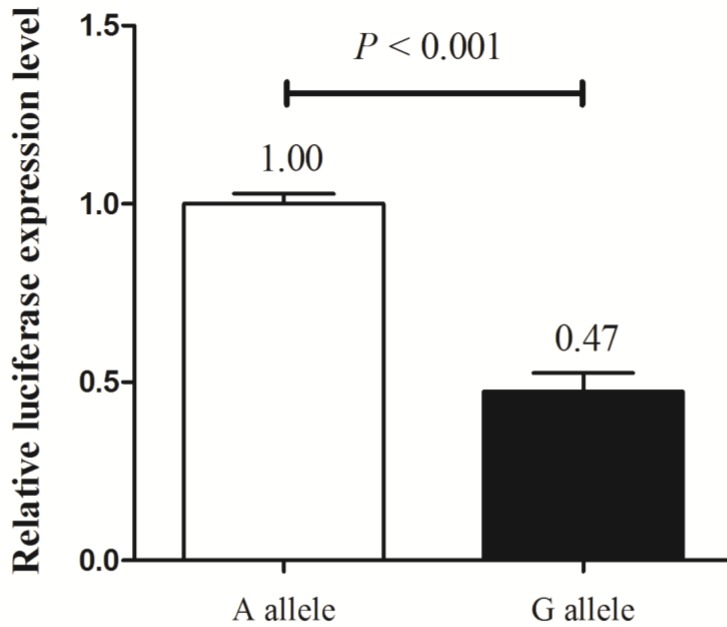
The effect of rs619586 on lnc MALAT1 transcriptional activity detected by luciferase assay.

Next, we explored the functional effects of different alleles of MALAT1 rs619586 by luciferase assay in TPC-1 cell line. After transfection, cells transfected with plasmid with G allele demonstrated a substantially decreasing trend of luciferase activity compared with those transfected with plasmid containing A allele (*P* < 0.001, fold change = 2.11, figure 2). Combined with the findings above, these results suggested that the A allele of rs619586 could directly influence MALAT1 expression in PTC.

### The different phenotypes of thyroid cancer cell line induced by MALAT1 polymorphism

**Figure 3:**
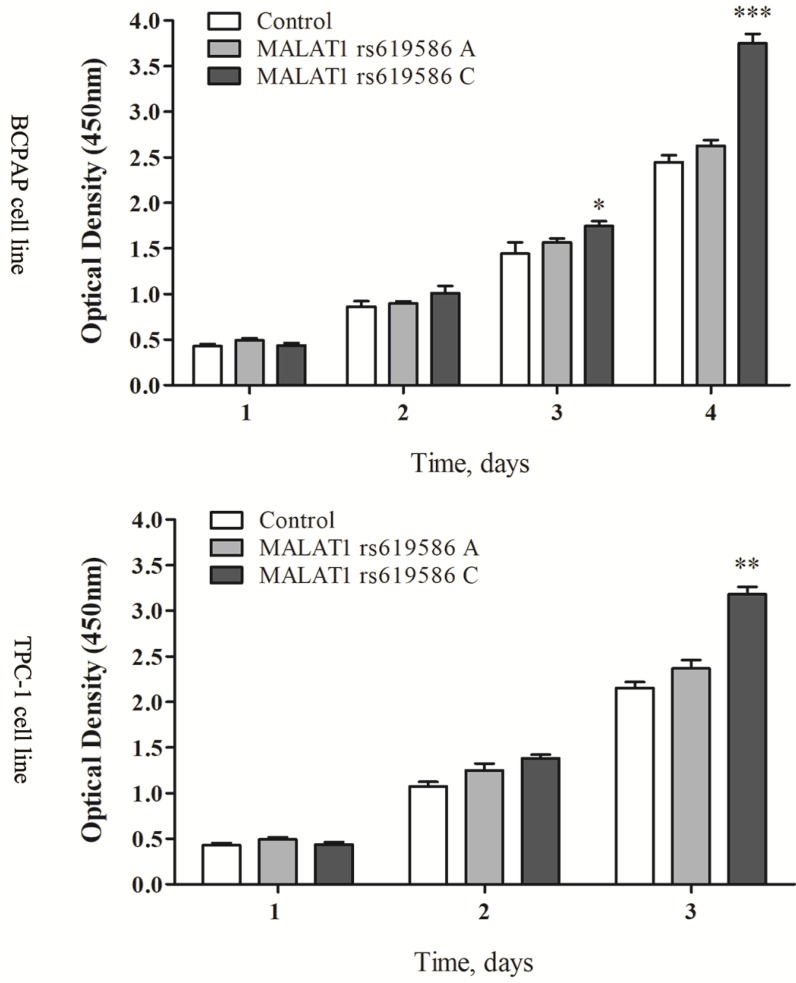
The lnc MALAT1 rs619586 influence the proliferation of PTC cells.

**Figure 4:**
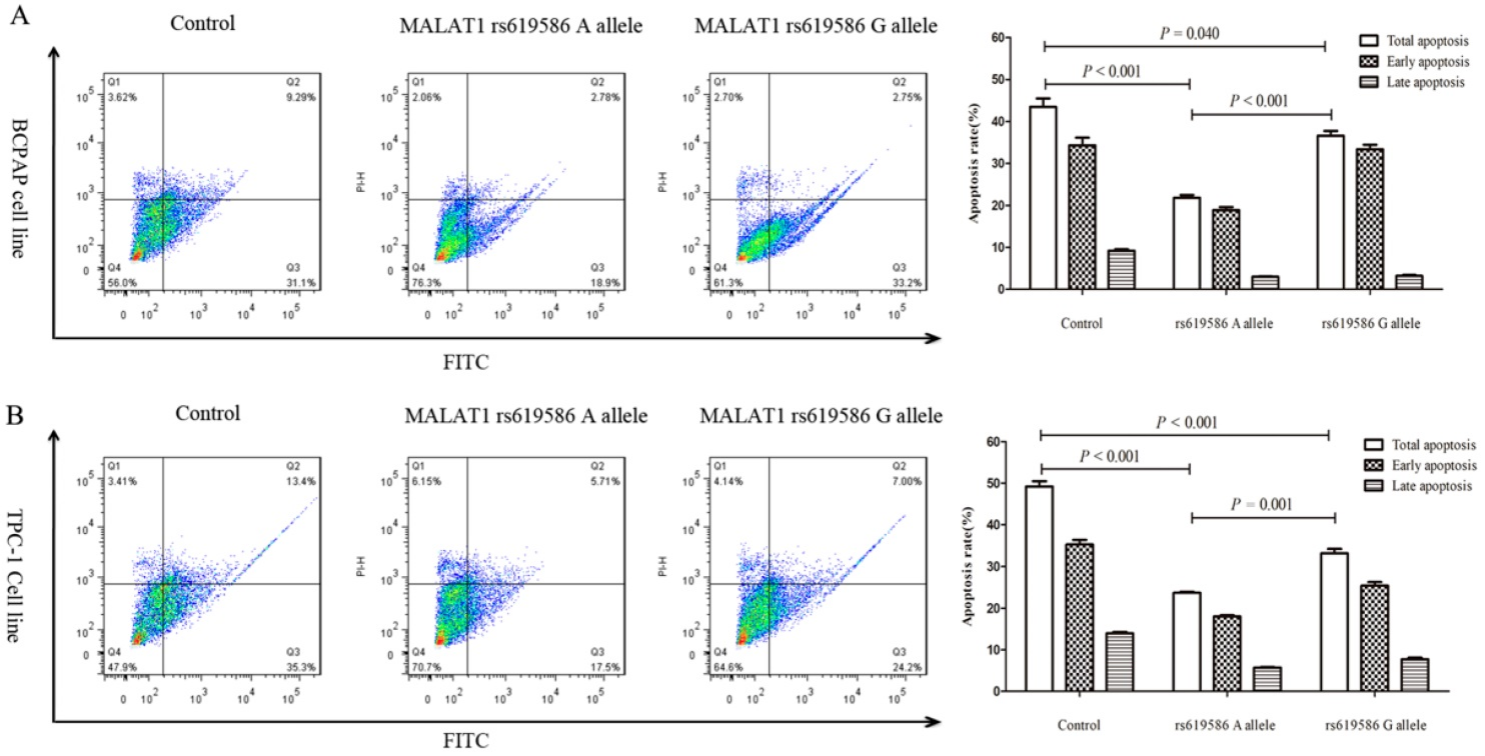
** The impact of lnc MALAT1 rs619586 on PTC cell apoptosis.** (A) the apoptosis of 10μg/ml cisplatin pre-treated BCPAP cell line; (B) the apoptosis of 10μg/ml cisplatin pre-treated TPC-1 cell line.

We also examined rs619586's effect on cell proliferation and apoptosis. Proliferation increased significantly in cells transfected with MALAT1 expression plasmid carrying A allele than G allele in the rs619586 site (figure 3), while the apoptosis capacity showed the opposite (figure 4). These findings further indicated SNP rs619586's molecular function and mechanism in papillary thyroid carcinogenesis.

### The EMT effects induced by different alleles of MALAT1 rs619586

**Figure 5:**
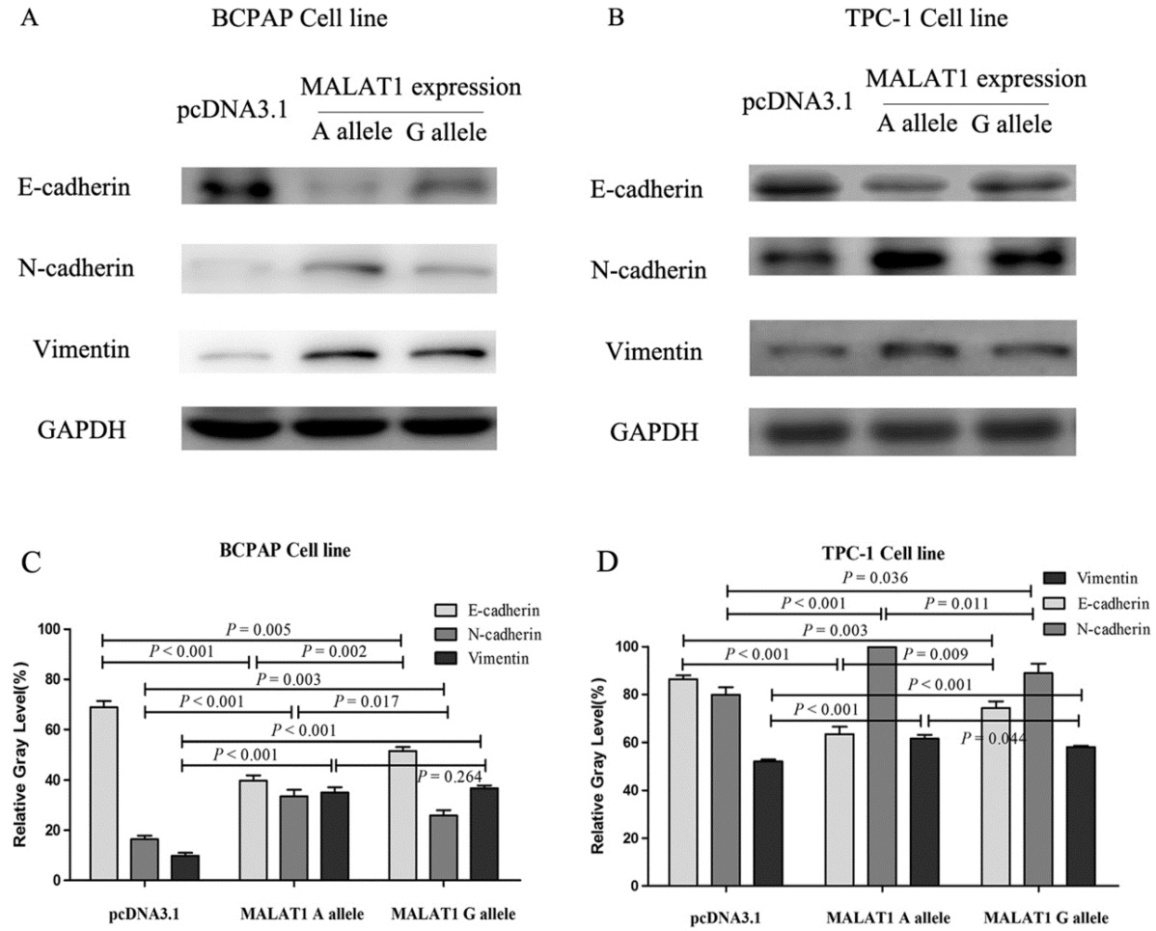
** The variations of EMT markers influenced by MALAT1 with different rs619586 polymorphism**. (A) the protein levels of E-cadherin, N-cadherin, and Vimentin in BCPAP cell line; (B) the protein levels of E-cadherin, N-cadherin, and Vimentin in TPC-1 cell line; (C) the corresponding relative gray assay level of (A); (D) the corresponding relative gray level of (B).

The EMT-related proteins were tested after transfection of different MALAT1 expression plasmids. The E-cadherin, N-cadherin and vimentin were chosen as the markers of EMT. The E-cadherin of cells transfected with MALAT1 expression plasmid carrying rs619586 A alllele was lower than that carrying G allele in both mRNA level and protein level, while N-cadherin and vimentin displayed diametric trends (figure 5).

## Discussion

In this study, we have systematically investigated the association between MALAT1 polymorphisms and TC (especially PTC) risk in a Chinese population. SNP rs619586 G allele was identified as a potential protective factor to reduce the risk of TC. We also demonstrated that rs619586 could influence cell proliferation and apoptosis, potentially via direct regulation on MALAT1 expression.

Overexpression of MALAT1 (metastasis associated lung adenocarcinoma transcript 1) was identified in different types of malignancies, including non-small cell lung cancer 21, ovarian cancer 22, prostate cancer 23 and other cancers 24, 25. Aberrant expression of MALAT1 was significantly associated with tumor invasion and metastasis, indicating this lncRNA could be a prognostic marker for cancers 26. In tumorigenesis, however, the categories of MALAT1 have still been less comprehensively investigated and understood, especially in TC. In a previous research, the MALAT1 expression was explored in tumor tissues and adjacent normal thyroid tissues in FTC patients. The average profile of MALAT1 in tumor cells was over 4-fold higher than in the corresponding normal cells 27. Besides, MALAT1 was also up-regulated in MTC patients, with an expression 150 times as much as normal thyroid tissues 28. In addition, our data in PTC patients indicated a high profile of MALAT1 in cancerous tissues. There was approximate 2-fold expression of MALAT1 in 64 PTC patients' cancerous tissues over normal tissues (Figure 1). These results confirmed MALAT1 overexpression in TC patients.

After decades of research on polymorphisms, several functional SNPs have been identified to involve in gene transcription and translation via different mechanisms 29, 30, and these regulations also exist in lncRNA expression 13, 31, 32. Our results also indicated SNP rs619586 triggered the increasing growth and suppressed apoptosis in thyroid cells. These findings demonstrated that the variants of rs619586 could directly induce the epithelial-to-mesenchymal transition (EMT) process of cells, an essential mechanism in cellular remodeling during carcinogenesis 33. Considering the promoter activity of different alleles of rs619586 and the corresponding variants of EMT markers, it is reasonable that this SNP trigger the EMT via up-regulating MALAT1 expression. These results were partially in accordance with the role of MALAT1 in EMT 34.

Recently, two independent GWAS researches about PTC in European populations. However, these studies did not list rs619586 in their results. For GWAS study usually remained the SNPs with *P* < 10^-8^ as remarkable results, it will lose information about other potential significant SNPs. Therefore, we downloaded the GWAS database of thyroid cancer and evaluated this SNP to seem whether this SNP was significant in European population. Unexpectedly, the SNP rs619586 did not display any significance in these European populations. Then we retrospected the MAF of SNP rs619586, the SNPs distributed differently in European population and in the Chinese population. Therefore, we believed the ethnic difference might lead to this problem.

Nevertheless, there are limitations in this study. One potential pitfall is that this study was based on a population enrolled from the hospital, therefore selection bias could be possible. Although we recruited over 1000 PTC patients and controls, the statistical power could be further improved in future large-scale study; replications of crowd tests in larger population and in population with different ethnicity might be helpful to make our results more convincing. Besides, tobacco consumption, alcohol consumption, and dietary preference were potential confounders for PTC but such information was partly missing in our study. In addition, because of the feature of the case-control study, our research could hardly display the final causal relationship effect of MALAT1 in stratified features.

## Conclusion

We identify that MALAT1 SNP rs619586 is associated with the risk of PTC in the Chinese population, and this polymorphism could directly reduce MALAT1 expression for the first time. We further demonstrate that SNP rs619586 could influence thyroid cell proliferation and apoptosis, indicating its potential role as a novel susceptibility for the papillary thyroid carcinogenesis.

## Supplementary Material

Supplementary table 1.

## Figures and Tables

**Table 1:** Demographic characteristics and clinical features

Variables	Cases (n =1134)			Controls (n = 1228)		*P*^a^
	n	%		n	%	
Age (years)						0.754
≤ 45	590	52.0		631	51.4	
> 45	544	48.0		597	48.6	
Sex						0.846
Male	355	31.3		389	31.7	
Female	779	68.7		839	68.3	
Topography						
T1	885	78.0				
T2	138	12.2				
T3	98	8.6				
T4	13	1.2				
Lymph Node						
N0	695	61.3				
N1	439	38.7				
Metastasis						
M0	1113	98.2				
M1	21	1.8				
Grade						
I + II	963	84.9				
III + IV	171	15.1				

^a^ Two-sided c^2^ test for the frequency distributions of selected variables between cases and controls.

**Table 2:** Genotyping of MALAT1 polymorphisms and their association with thyroid cancer.

SNPs	Genotypes	Cases			Controls		*P*^a^	Adjusted OR
		n = 1134	%		n = 1228	%		(95% CI)^b^
rs11227209	CC	1042	91.97		1110	90.46		Reference
	CG	91	8.03		110	8.96	0.389	0.88 (0.66-1.18)
	GG	0	0		7	0.57	0.962	NA
	Additive model						0.09	0.79 (0.60-1.04)
	Dominant model						0.196	0.83 (0.62-1.10)
	Recessive model						0.962	NA
rs619586	AA	1002	88.36		1051	85.59		Reference
	AG	131	11.55		167	13.6	0.116	0.82 (0.64-1.05)
	GG	1	0.09		10	0.81	**0.032**	**0.11 (0.01-0.82)**
	Additive model						**0.017**	**0.76 (0.60-0.95)**
	Dominant model						**0.045**	**0.78 (0.61-0.99)**
	Recessive model						**0.033**	**0.11 (0.01-0.84)**
rs3200401	CC	808	71.32		872	71.18		Reference
	CT	302	26.65		322	26.29	0.878	1.01 (0.84-1.22)
	TT	23	2.03		31	2.53	0.424	0.80 (0.46-1.38)
	Additive model						0.761	0.98 (0.83-1.14)
	Dominant model						0.944	0.99 (0.83-1.19)
	Recessive model						0.411	0.80 (0.46-1.37)

^a^ Two-sided χ^2^ test. ^b^ Adjusted for age and sex in logistic regression model.

**Table 3:** Stratification analysis of MALAT1 rs619586 in thyroid cancer in a dominant model

Variables	AA (case/control)			AG/GG (case/control)			*P*^a^	Adjusted OR
	n	%		n	%			(95% CI)^b^
Age (years)								
≤45	523/533	88.64/84.47		67/98	11.36/15.53		0.035	0.70 (0.50-0.98)
>45	479/518	88.05/86.77		65/79	11.95/13.23		0.525	0.89 (0.63-1.27)
Sex								
Male	322/333	90.70/85.60		33/56	9.30/14.40		0.036	0.61 (0.39-0.87)
Female	680/718	87.29/85.58		99/121	12.71/14.42		0.316	0.86 (0.65-1.15)
Topography								
T1	783/1051	88.47/85.59		102/177	11.53/14.41		0.054	0.77 (0.60-1.01)
T2	121/1051	87.68/85.59		17/177	12.32/14.41		0.531	0.84 (0.49-1.44)
T3	88/1051	89.80/85.59		10/177	10.20/14.41		0.235	0.67 (0.34-1.31)
T4	10/1051	76.92/85.59		3/177	23.08/14.41		0.403	1.74 (0.47-6.43)
Lymph Node								
N0	613/1051	88.20/85.59		82/177	11.80/14.41		0.116	0.80 (0.60-1.06)
N1	389/1051	88.61/85.59		50/177	11.39/14.41		0.104	0.76 (0.54-1.06)
Metastasis								
M0	985/1051	88.50/85.59		128/177	11.50/14.41		0.036	0.77 (0.60-0.98)
M1	17/1051	80.95/85.59		4/177	19.05/14.41		0.447	1.54 (0.51-4.67)
Grade								
I + II	851/1051	88.37/85.59		112/177	11.63/14.41		0.051	0.78 (0.60-1.00)
III + IV	151/1051	88.30/85.59		20/177	11.70/14.41		0.37	0.80 (0.49-1.81)

^a^ Two-sided χ^2^ test. ^b^ Adjusted for age and sex in logistic regression model.
