# Supplementary material for: Effect of MALAT1 Polymorphisms on Papillary Thyroid Cancer in a Chinese Population
Source: J Cancer. 2019 Sep 19;10(23):5714–21. doi: 10.7150/jca.28887 (PMC6879318; doi:10.7150/jca.28887)
Supplement: Supplementary file 1 — Supplementary table 1. [file jcav10p5714s1.pdf]

Supplementary Table 1. Primers for SNP detection

Name	Sequence (5'-3')	Base
rs11227209-F	TGGGACGCAGCGACGAGTT	19
rs11227209-R	TCCAAACCCCAAGACCAAACT	21
rs11227209-P-C	FAM- CCTTATAGGCTGGCCAT-MGB	17
rs11227209-P-G	HEX- CCTTATAGGGTGGCCAT-MGB	17
rs619586-F	TGCGTAATGGAAAGTAAAG	19
rs619586-R	GGTCATCAAACACCTCAC	18
rs619586-P-A	FAM- GTAAACTATACCTACTGTC-MGB	19
rs619586-P-G	HEX- GTAAACTATACCTGCTGTC-MGB	19
rs3200401-F	ATTTGGAGGGATGGGAG	17
rs3200401-R	TTCTAATAGCAGCGGGAT	18
rs3200401-P-C	FAM- GACTTCAGGTCTGTCTG-MGB	17
rs3200401-P-T	FAM- GACTTCAGGTTTGTCTG-MGB	17
